# Characterization of a Novel Dengue Serotype 4 Virus-Specific Neutralizing Epitope on the Envelope Protein Domain III

**DOI:** 10.1371/journal.pone.0139741

**Published:** 2015-10-02

**Authors:** Guang-Hui Ji, Yong-Qiang Deng, Xiao-Jie Yu, Tao Jiang, Hua-Jing Wang, Xin Shi, Da-Peng Zhang, Xiao-Feng Li, Shun-Ya Zhu, Hui Zhao, Jian-Xin Dai, Cheng-Feng Qin, Ya-Jun Guo

**Affiliations:** 1 Department of Traditional Chinese Medicine, Navy General Hospital, Beijing, China; 2 International Joint Cancer Institute, Second Military Medical University, Shanghai, China; 3 State Key Laboratory of Pathogen and Biosecurity, Beijing Institute of Microbiology and Epidemiology, Beijing, China; Institut Pasteur of Shanghai, CHINA

## Abstract

The dengue virus (DENV) envelope protein domain III (ED3) has been suggested to contain receptor recognition sites and the critical neutralizing epitopes. Up to date, relatively little work has been done on fine mapping of neutralizing epitopes on ED3 for DENV4. In this study, a novel mouse type-specific neutralizing antibody 1G6 against DENV4 was obtained with both prophylactic and therapeutic effects. The epitope was mapped to residues 387–390 of DENV4 envelope protein. Furthermore, site-directed mutagenesis assay identified two critical residues (T388 and H390). The epitope is variable among different DENV serotypes but is highly conserved among four DENV4 genotypes. Affinity measurement showed that naturally occurring variations in ED3 outside the epitope region did not alter the binding of mAb 1G6. These findings expand our understanding of the interactions between neutralizing antibodies and the DENV4 and may be valuable for rational design of DENV vaccines and antiviral drugs.

## Introduction

Dengue is the most important arbovirus disease in tropical and subtropical countries. Clinical symptoms range from a self-limited, acute, febrile disease called dengue fever (DF) to severe dengue hemorrhagic fever (DHF), and dengue shock syndrome (DSS)[1]. It was estimated that over 2.5 billion people are at risk of contracting dengue, and that about 390 million people are infected with dengue every year, resulting in 100 million symptomatic infections with 250,000 cases of DHF/DSS per year worldwide [2–4].

Dengue viruses (DENV) are composed of four genetically and antigenically related viruses termed DENV1-4 [5]. They have a relatively simple enveloped virion that is 50 nm in diameter and consist of a capsid protein (C), membrane protein (M), and a major envelope glycoprotein (E). The E protein ectodomain can be divided into three structural domains designated domain I, domain II, and domain III (ED1, ED2, and ED3), respectively. ED1 is a central, eight stranded β-barrel, which contains a single N-linked glycan in most DENV strains. ED2 is a long, finger-like protrusion from ED1 with a highly conserved fusion peptide (residues 98–110) at its distal end and mediates post-entry endosomal fusion [6–8], it contains the major flavivirus group and subgroup cross-reactive epitopes [9–11]. ED3 adopts an immunoglobulin-like fold and is characteristic of many cell receptors [12]. In addition, ED3 contains the critical and dominant virus subcomplex and type-specific neutralization sites [13–16].

Dengue vaccine development has been hampered by concerns that cross-reactive antibodies elicited by a candidate vaccine could increase the risk of development of more severe clinical forms [17]. One possible strategy to reduce risks associated with a dengue vaccine is the development of a vaccine composed of selected specific critical neutralizing epitopes of each of the serotypes.

The most potent neutralizing mAbs were reported to bind to ED3 [18–20]. A more thorough analysis of DENV ED3 neutralizing epitopes will provide a better understanding of the molecular mechanism of DENV neutralization and aid in the development of candidate DENV vaccines and antibody therapy. In previous studies, a great many DENV type-specific, sub-complex and complex neutralizing epitopes have been identified on ED3 for DENV1-4 [15,21–29]. Of all these neutralizing mAbs, serotype-specific mAbs were reported to have the greatest neutralizing activity [22,30]; furthermore, type-specific neutralizing antibodies might have low risk of inducing infection enhancement of other DENV serotypes [24,31]. However, to our knowledge, relatively few work has been reported on fine mapping of type-specific neutralizing epitopes for DENV4 [29].

In this study, a novel DENV4 type-specific monoclonal antibody specific to ED3, designated mAb 1G6, was generated and found to have potent neutralizing and protective activities. The neutralizing epitope was then mapped to motif ^386^ALTLH^390^ by phage-display technique with two critical residues (T388 and H390) identified. These results indicated that the DENV4 type-specific neutralizing mAb may be useful for both type-specific diagnosis and immunotherapy and may provide further insights into the mechanisms underlying DENV infection.

## Materials and Methods

### Ethics Statements

The animal experiments were approved by the Experimental Animal Ethic and Welfare Committee of Beijing Institute of Microbiology and Epidemiology. The use of human sera in this study was complied with the Ethical Standards of the Committee on Publication Ethics.

### Cells and viruses

BHK21 cells were maintained in Dulbecco’s Modified Essential Medium (DMEM) supplemented with heat-inactivated 10% fetal bovine serum (FBS) (PAA) and antibiotics with 1% penicillin G and 1% streptomycin [9]. Mosquito C6/36, mouse myeloma SP2/0 and hybridoma cells were grown in RPMI 1640 medium supplemented with 10% FBS and antibiotics. Media were purchased from Invitrogen. All cells were maintained in a 5% CO2 incubator at 37°C, except for C6/36 cells, which were maintained at 28°C. DENV1 strain 128 (GenBank FJ176780), DENV2 strain 43 (GenBank AF204178), DENV3 strain 80–2 (GenBank AF317645), DENV4 strain B5 (GenBank AF289029) and DENV4 strain H241 (GenBank U18433) were propagated in C6/36 cells by using RPMI 1640 medium and the titers were measured by standard plaque forming assay in BHK–21 cells.

### Construction, Expression and purification of respective and tandem ED3 proteins

The cDNA encoding DENV1-4 ED3 was amplified by polymerase chain reaction (PCR) by using RNA from four dengue virus serotypes as templates and tandem ED3 (tED3) was constructed by connecting four ED3s using a flexible (G4S)_3_ linker described previously [32]. All these products were cloned into the EcoR I and BamH I site of the expression vector pGEX4T-2. After confirmation of the sequence by sequencing, Escherichia coli cells (strain BL21 (DE3)) (Novagen) were transformed with the pGEX-4T-2 vector containing the respective ED3 of DENV1-4. Protein expression was subsequently induced by adding isopropy-β-D-thiogalactoside (IPTG) to a final concentration of 0.8mM when the bacteria culture reached an OD_600 nm_ of 0.5, and further incubated at 16°C for 4 h. The cells were then harvested by centrifuging at 9000×g for 15 min at 4°C and resuspended in lysis buffer before sonication. After centrifugation, the supernatant was subjected to GST affinity purification (Bio-Rad) and the fusion proteins were eluted with a buffer containing 10 mM GSH. Concentrations of the purified proteins were determined by BCA kit (Pierce). Western blot analysis was performed using a polyclonal antibody against the GST-tag to determine the authenticity of the proteins.

### Generation and purification of mAbs against respective DENV1-4

Six-week-old female BALB/c mice were immunized intraperitoneally with 100 μg of tED3 protein mixed with an equal volume of complete Freund’s adjuvant (Sigma). Mice were boosted three times with 100 μg protein in incomplete Freund adjuvant at 2-week intervals. Mice were bled from the retro-orbital plexus to check antibody production. Mice received a final boost with 100 μg of tED3 in phosphate-buffered saline (PBS) intravenously. Three days later splenocytes were fused to mouse myeloma SP2/0 cells and hybridomas secreting anti-DENV antibodies were generated according to standard procedures described elsewhere [33]. Hybridoma colonies were screened by ELISA for mAbs that bound with tED3. Selected clones were subcloned by limiting dilution, isotyped (Southern Biotech), and purified using protein A affinity chromatography (Invitrogen).

### Indirect immunofluorescence assay

The immunofluorescence assay (IFA) was performed as described elsewhere [32]. Briefly, BHK21 cells were passaged in RPMI–1640 medium containing 10% FBS and then infected with different dengue virus serotypes. Cells were harvested 3–4 days post-infection, and resuspended in RPMI 1640 containing 10% FBS. The resuspended cells were then dropped onto slides and incubated for 6 h at 37°C and 5% carbon dioxide. Following this, the slides were rinsed twice in PBS and fixed with acetone for 30 min in -20°C. The slides were incubated with100-fold dilution of mAb 1G6 or normal ascites fluid. After incubation for 1 h at 37°C, the slides were washed five times in PBS and dried at room temperature. Then, fluorescein isothiocyanate (FITC)–conjugated goat anti-mouse IgG was added and incubated for another 1 h at 37°C. After being washed five times in PBS, positive cells were detected using a fluorescent microscope.

### Indirect enzyme-linked immunosorbent assay (ELISA)

The wells of 96-well microtiter plates were coated with 50 μl /well of serial dilutions of recombinant DENV1-4 proteins overnight. After incubation with 10% skimmed milk in PBS in each well for 2 h at 37°C to prevent nonspecific binding, 50 μl each of 1:100 diluted antibodies was added to each well and incubated for 1 h at 37°C. The DENV antisera and normal mice sera were used as the positive and negative control, respectively. After samples were washed 5 times with PBS containing Tween 20 (PBST) (0.1%), horseradish peroxidase (HRP)–conjugated goat anti-mouse immunoglobulin G (IgG) (KPL) was added. Following this, samples were washed 5 times with PBST (0.1%). Then, 50 μl of TMB One solution (Promega) was added and incubated for 20 min. After termination by adding 50 μl of 2M sulfuric acid, the absorbance at 450nm and 630nm was measured on an ELISA reader. In this manner, the amount of each protein that gave a similar optical density was standardized and selected for antigen coating.

### Plaque reduction neutralization test (PRNT)

Various concentrations of mAbs were mixed with the same volume of 100 plaque-forming units (p.f.u.) of DENV1-4 and incubated at 37°C for 1 h. Then the virus-antibody mixture were added to a monolayer of BHK–21 cells in a 6-well plate and incubated for 1 h at 37°C. Supernatants were removed and 3 ml of 1.2% (w/v) LMP agarose (Promega) in 2×DMEM plus 2% (v/v) FBS was laid onto the infected cells. After further incubation at 37°C for 4 to 7 days, the wells were fixed and stained with crystal violet to visualize the plaques. PRNT_50_ values were determined using non-linear regression analysis [22].

### Mouse experiments

The neutralization experiment *in vivo* was done using one-day-old suckling mice as previously described [32]. Groups of suckling mice were randomised with 9–11 mice in each group. In prophylaxis experiments, suckling mice were administered a single 50 μg dose of mAb 1G6 intraperitoneally (IP) one day before infection. Subsequently, mice were challenged with 10^5^ PFU/ml DENV4 by an intracerebral route. In post-exposure therapeutic experiments, a single 50 μg dose of mAb 1G6 was administered IP four or twenty-four hours after infection with 10^5^ PFU/ml DENV4. Mice were observed daily and the mortality and morbidity were recorded for three weeks. Protection significance was evaluated using log-rank analysis of a Kaplan-Meier survival curve compared to the controls.

Mice exhibiting signs of severe disease with ruffled fur, hunched posture and moribund lethargy were euthanized by cervical dislocation and counted as being dead on the following day. The survived mice at the end of the experiments were also euthanized and counted as survival. All operations were performed to minimize animal suffering.

### Pre- and post-adsorption inhibition assay

Neutralization of DENV4 before or after virus adsorption to BHK21 cells (1 h at 4°C) was performed as described previously [34]. Briefly, for the pre-adsorption assay, 0.3 ml of a virus dilution containing 50 PFU/well was mixed with the same volume of 5-fold mAb dilutions, and the mixture was incubated for 1 h at 4°C. The virus plus mAb mixture was then added to cells (80 to 90% confluent), and incubation continued for an additional hour at 4°C, a temperature that only allows virus adsorption to occur. While in the post-adsorption, 0.3 ml of the virus seed dilution from the pre-adsorption assay was mixed with 0.3 ml of PBS and added directly to cells, and the mixture was incubated for 1 h at 4°C. Unadsorbed virus was removed by three washes with PBS at 4°C. The 5-fold MAb dilutions were then added directly to wash cells containing adsorbed virus, followed by incubation for 1 h at 4°C. Negative controls received 0.3 ml of PBS instead of antibody. All cells were washed and an agarose overlay was added. Four to six days later, plaques were scored after fixation and staining with crystal violet.

### Western blot

Recombinant proteins were resolved on reducing 10% SDS polyacrylamide gels and transferred to nitro-cellulose membranes. For immunoblot, membranes were blocked with 10% skimmed milk in PBST for 2 h at room temperature (RT), washed three times in PBST and reacted with mAb 1G6 at the concentration of 1μg/ml in 10% skimmed milk in PBST for 1 h at RT. After washing three times, membranes were incubated with peroxidase-conjugated goat anti-mouse IgG (Amersham) at a 1:5000 dilution in PBST for 1 h at RT. Then membranes were washed again and developed with chemiluminescence reagents (ECL, Amersham).

### Epitope mapping by 12mer phage-displayed random peptide library

The conformational requirement of the epitope was first determined by Western blot as described above with and without reduction with 2-mercaptoethanol.

The epitope of mAb 1G6 was then determined using the phage-display peptide library kit according to the manufacturer’s instructions (New England BioLabs). Briefly, purified 1G6 (100 μg/ml) was immobilized on ELISA plates to select immunopositive phage clones, and bounded phage clones were selected after biopanning three times. The immunopositive phage clones were further characterized by DNA sequencing and peptide sequences were then aligned.

### Sequential truncations and site-directed mutagenesis

To further confirm the phage-displayed epitope, sequential truncations of the DENV4 rED3 from the C termini by 5aa intervals were expressed in Escherichia coli (*E*. *coli*) cells (strain BL21 (DE3)) as described above.

To further identify the critical residues involved in the epitope recognized by mAb 1G6, a total of 5 single amino acid substitutions covering 386-390aa and a combination of substitutions (T388 and H390) were introduced by site-directed mutagenesis, based on the above results and the crystallization of DENV4 ED3. All residues were subjected to glycine scanning mutagenesis, essentially to remove the side chain of each residue. For studies on different genotypes of DENV4, ED3 sequences of strain Brazil 1982 (Genotype II, from Brazil 1982) and strain ThD4-0017-97 (Genotype III, from Thailand 1997) variable amino acids of ED3 sequence were used.

### Affinity measurements by indirect ELISA

The binding affinity was determined by titration in an indirect ELISA (see above) with the different rED3 mutants and compared to the wild-type DENV4 rED3. Binding curves and KD values were determined by non-linear regression analysis.

### Sequence alignment and molecular modeling

Nucleotide and deduced amino acid sequences encoding the ED3 genes of DENV1-4 tested were obtained from GenBank and aligned. The NCBI accession numbers were described above. Based on the crystal structures of DENV1-4 E protein (PDB code: 3IRC, 2JSF, 1UZG and 2H0P, respectively), the comparison of four DENV ED3 crystal structures was done using Insight II 2005 (Accelrys). The location and structure of the 1G6 epitope on DENV4 ED3 crystal structure was also analyzed.

## Results

### Characterization of a DENV4-specific monoclonal antibody

**Fig 1:**
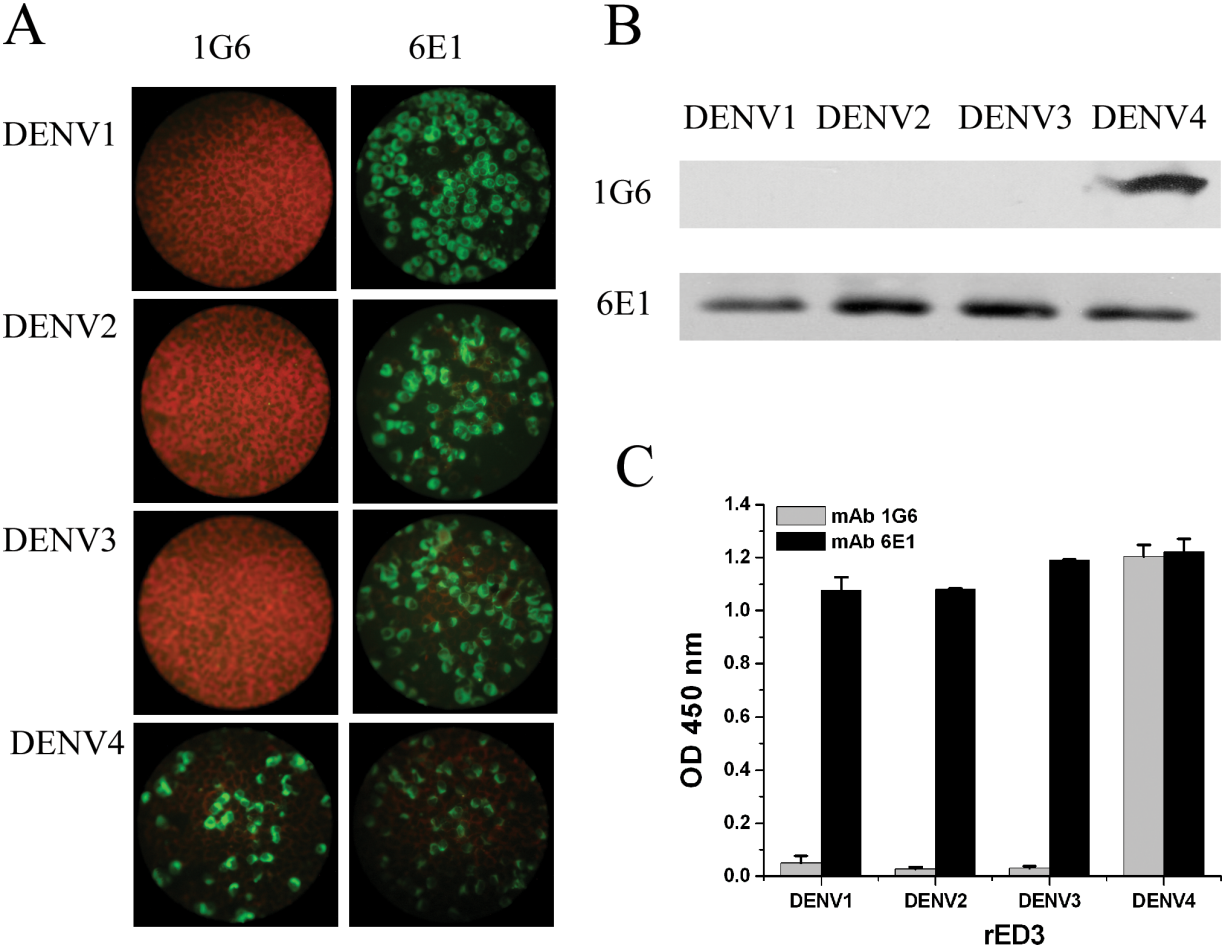
Characterization of mAb 1G6 *in vitro*. **(A)** Binding of mAb 1G6 with DENV1-4 by IFA. DENV antigen was detected on day 3 after infection with DENV1-4 with mAb 1G6 and an FITC-conjugated secondary antibody. Cells were counterstained with Evan’s blue, resulting in red fluorescence of cells. **(B)** Binding of mAb 1G6 with purified DENV1-4 ED3 proteins by western blotting. The cross-reactive mAb 6E1 was used as a positive control. **(C)** Binding graph of mAb 1G6 with purified DENV1-4 ED3 proteins by ELISA.

A total of 14 mAbs against different DENV serotypes were obtained by both ELISA and IFA (S1 Fig and S1 Table). Amongst these mAbs, 1G6 recognized only the viral proteins of DENV4 infected cells by IFA (Fig 1A). Meanwhile, this antibody did not cross-reacted with other related flaviviruses including WNV, JEV, YFV and TBEV by IFA (data not shown). Indirect ELISA and Western blot based on DENV1-4 rED3s further confirmed that mAb 1G6 only reacted with DENV4 ED3 protein (Fig 1B and 1C). Meanwhile, Treatment of rDENV4 ED3 with sodium dodecyl sulfate (SDS) and 2-mercaptoethanol had no apparent effect on binding to mAb 1G6 as determined by Western blot analysis (data not shown). These result suggested that mAb 1G6 is a DENV4 type-specific antibody which recognized a linear epitope on DENV4 ED3.

### Mechanism of 1G6-mediated *in vitro* neutralization

**Fig 2:**
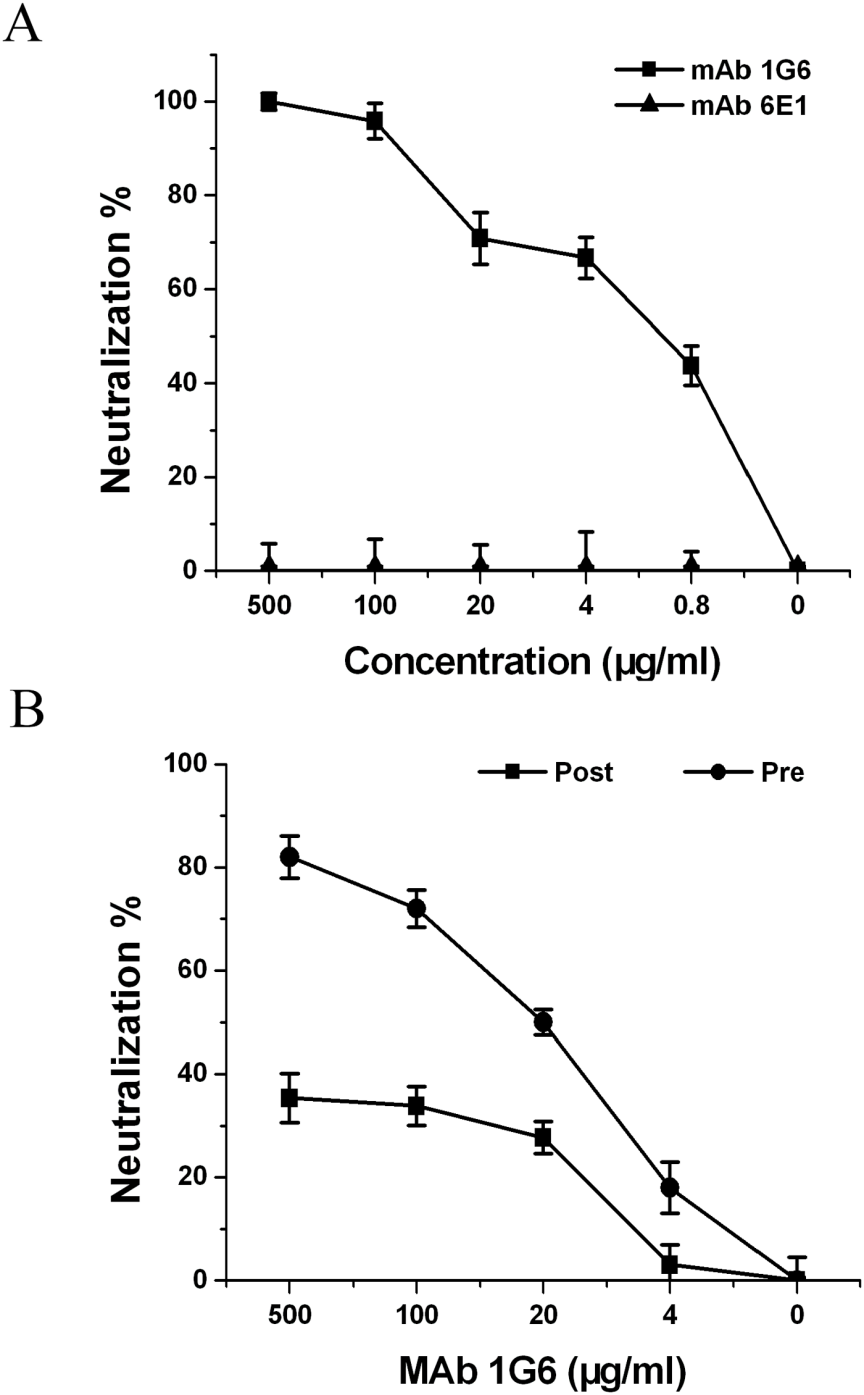
MAb 1G6 mediated neutralization in vitro through blocking viral adsorption. MAbs with concentrations of 500, 100, 20, 4, 0.8μg/ml were mixed with constant 50 PFU viruses. The results were expressed as percentage of neutralization (% Neutralization) shown with standard deviations. **(A)** Neutralizing ability of mAb 1G6 against DENV4. The non-neutralizing mAb 6E1 was used as a negative control. **(B)** Pre- and post-adsorption inhibition assays. Pre-binding of DENV4 with 1G6 significantly protected against infection (P<0.001) (Pre).

The neutralizing ability of mAb 1G6 against DENV4 infection was determined by PRNT assay. The result showed that mAb 1G6 effectively neutralized DENV4 infection on BHK21 cell with the PRNT_50_ concentration at 1.52 ± 0.63 μg/ml (Fig 2A). Then, a standard pre- and post-adsorption inhibition assay was performed to further define the mechanism of 1G6-mediated neutralization. The results showed that pre-binding with 1G6 inhibited infection more efficiently (p<0.001), indicating that mAb 1G6 neutralizes DENV4 infection primarily at a step before DENV4 adsorption (Fig 2B).

### Prophylactic and therapeutic efficacy of mAb 1G6

**Fig 3:**
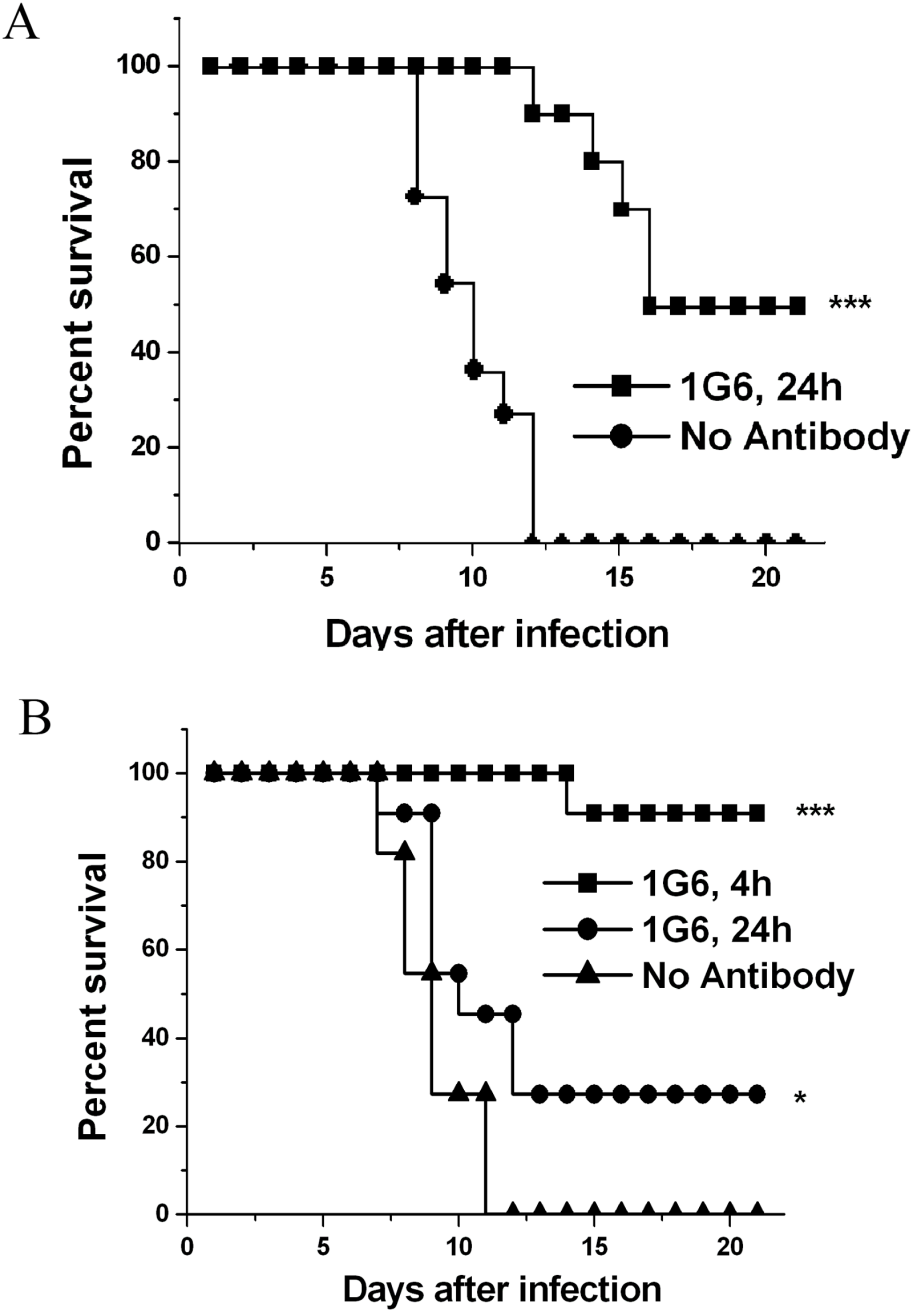
Prophylactic and therapeutic efficacy of 1G6 in suckling mice. **(A)** Mice were administered saline or a single 50 μg dose of 1G6 via an IP route one day prior to infection and then infected intracerebrally with 10^5^ PFU/ml of the mouse-adapted DENV–4 virus and monitored for survival for 21 days after infection. **(B)** Therapeutic efficacy of 1G6 was tested by administering a single 50 μg dose of 1G6 intraperitoneally 4h or 24h after infection with 10^5^ PFU/ml intracerebrally. The number of suckling mice for each group ranged from 10 to 11. Kaplan—Meier survival curves were analyzed by the log-rank test and significant differences are indicated by asterisks (*** p<0.001; * p<0.05).

**Fig 4:**
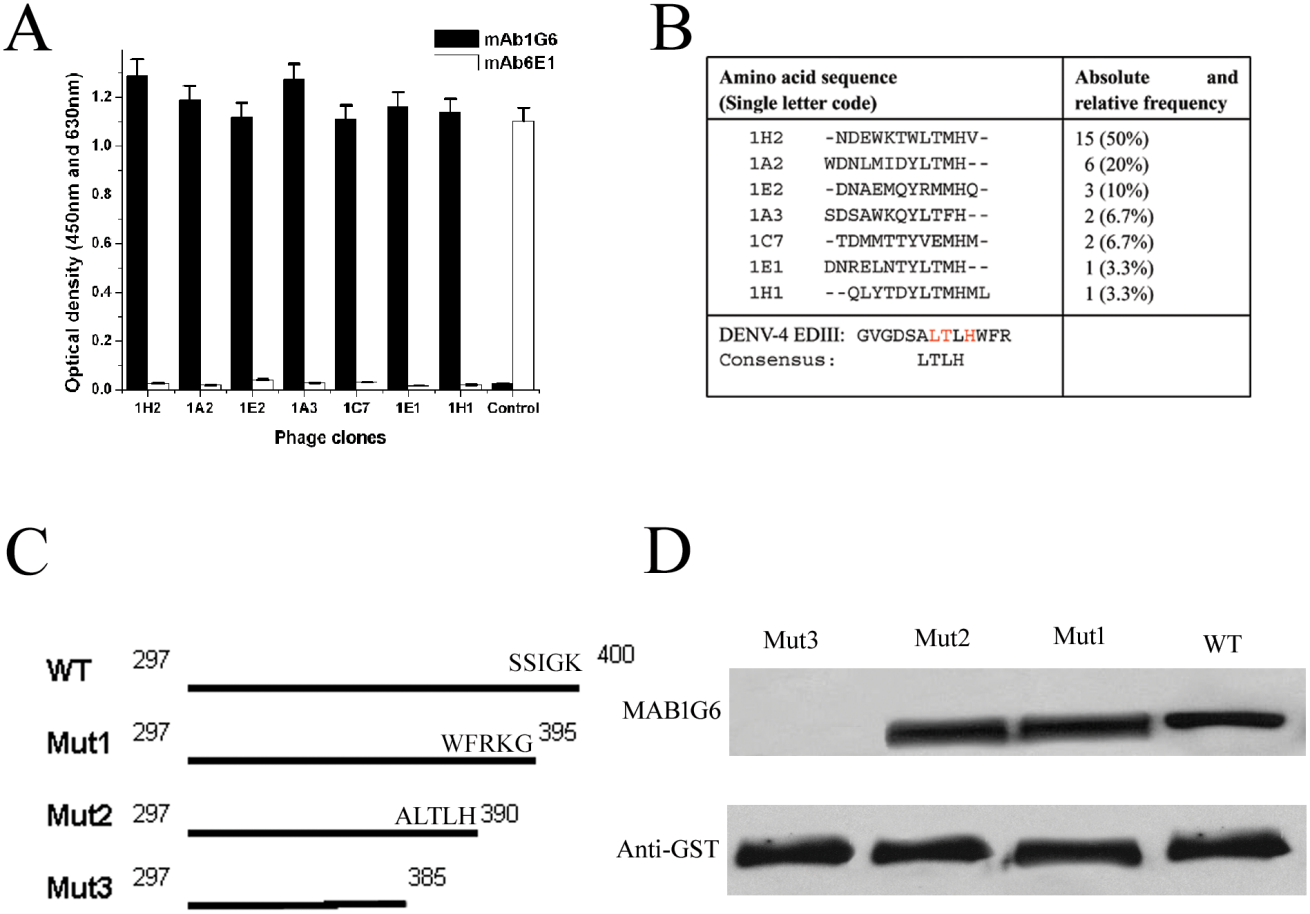
Epitope mapping of 1G6. **(A)** Unique mAb 1G6-specific phages after three rounds of bio-panning. The binding of isolated phage clones to 1G6 was examined by ELISA. Data represent the mean ± S.D. of duplicate measurements. **(B)** Peptide insert sequences (single letter code) enriched after three rounds of selection. Selected peptide sequences are aligned for the consensus motif which is highlighted in bold letters. Binding of 1G6 to control phage clone and binding of control mAb 6E1 with 1G6-specific phage particles were included as specificity controls. Absolute frequency means the times that a specific phage-displayed peptide was identified; while relative frequency means the proportion of a specific phage-displayed peptide normalized by the total number of phage clones. **(C)** Schematic of generating truncated rED3 proteins. **(D)** Western blot of purified wild type and DENV4 rED3 truncations. The polyclonal antibody anti-GST was used as a positive control.

Prophylactic and therapeutic efficacy of mAb 1G6 was also investigated in mice by administering a single 50 μg dose of mAb 1G6 or PBS before or after lethal DENV4 challenge. The results showed that prophylactic administration with a single dose of 50 μg 1G6 24 h before lethal DENV4 challenge conferred 50% protection in mice (p<0.001) (Fig 3A). Notably, 90% of mice survived when 1G6 was administered 4 h after challenge (P < 0.001), even 30% of mice survived when given 24 hours after DENV4 challenge (P < 0.05) (Fig 4B). These results strongly indicated the therapeutic potential of 1G6 in antibody based therapy against DENV4 infection.

### The motif ^386^ALTLH^390^ of DENV4 E protein is critical for 1G6 binding

The epitope of mAb 1G6 was mapped using a 12mer phage-displayed random peptide library. After three rounds of panning, a total of seven unique sequences were obtained (Fig 4A). The consensus sequences containing the YLTMH motif was aligned with the sequence ^381^GVGDSALTLHWFR^393^ on ED3 of DENV4 (Fig 4B).

To test the dependency of the phage-displayed epitope, sequential truncations of the DENV4 rED3 from the C termini by 5 aa intervals were expressed in *E*. *coli* cells as described above (Fig 4C). When amino acids ranging from either 391–395 aa or 396–400 aa of E protein were deleted, binding activity of mAb 1G6 with the ED3 protein have no apparent influence by both ELISA and Western blot analysis. However, deletion of the amino acids involving 386–390 aa resulted in the loss of binding. On the contrary, recovering the 5 aa residues could restore its binding ability (Fig 4D). These results showed that the epitope of 1G6 located within 386–390 aa of the DENV4 E protein.

### T388 and H390 are critical amino acids recognized by mAb 1G6

**Fig 5:**
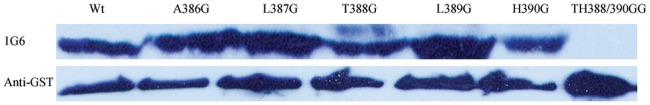
Western blot of 1G6 binding to rED3 with different mutations. Simultaneous mutations of T388 and H390 abrogated the binding of 1G6 to rED3.

The importance of residues for mAb binding was analyzed using an indirect ELISA with each of the 7 rED3 mutated proteins as previously described [21]. As shown in Table 1, the affinity were reduced by 11.7 and 10.5 times respectively when amino acids T388 and H390 were substituted by glycine, while the L389G mutation resulted in only 1.1 times reduction of affinity. Furthermore, simultaneous mutation of T388 and H390 completely abrogated mAb 1G6 binding, which indicating that these two amino acids have synergistic action. Western blot analysis also showed the loss of binding to the rED3 combination mutation (Fig 5). These results indicated that T388 and H390 were critical residues for the 1G6 binding.

**Table 1:** Amino acid sequences of DENV-4 ED3 mutants and relative dissociation constants compared to wild-type (Wt) rED3.

Proteins	Sequence	MAb 1G6	MAb 6E1
Wt	386ALTLH390	1.0	1.0
A386G	386GLTLH390	1.1	1.0
L387G	386AGTLH390	0.9	1.1
T388G	386ALGLH390	**11.7**	1.0
L389G	386ALTGH390	1.1	0.9
H390G	386ALTLG390	**10.5**	0.9
TH388/390GG	386ALGLG390	**NB**	0.9
Mut 3	386 - - - - - 390	**NB**	1.1

Note: The mutated amino acids are underlined; “-” indicates residues deletion; “NB” means not binding.

Significant decreases in binding affinity relative to Wt from genotype I are shown in bold.

### Sequences alignment and comparison of crystal structures of DENV1-4 ED3

**Fig 6:**
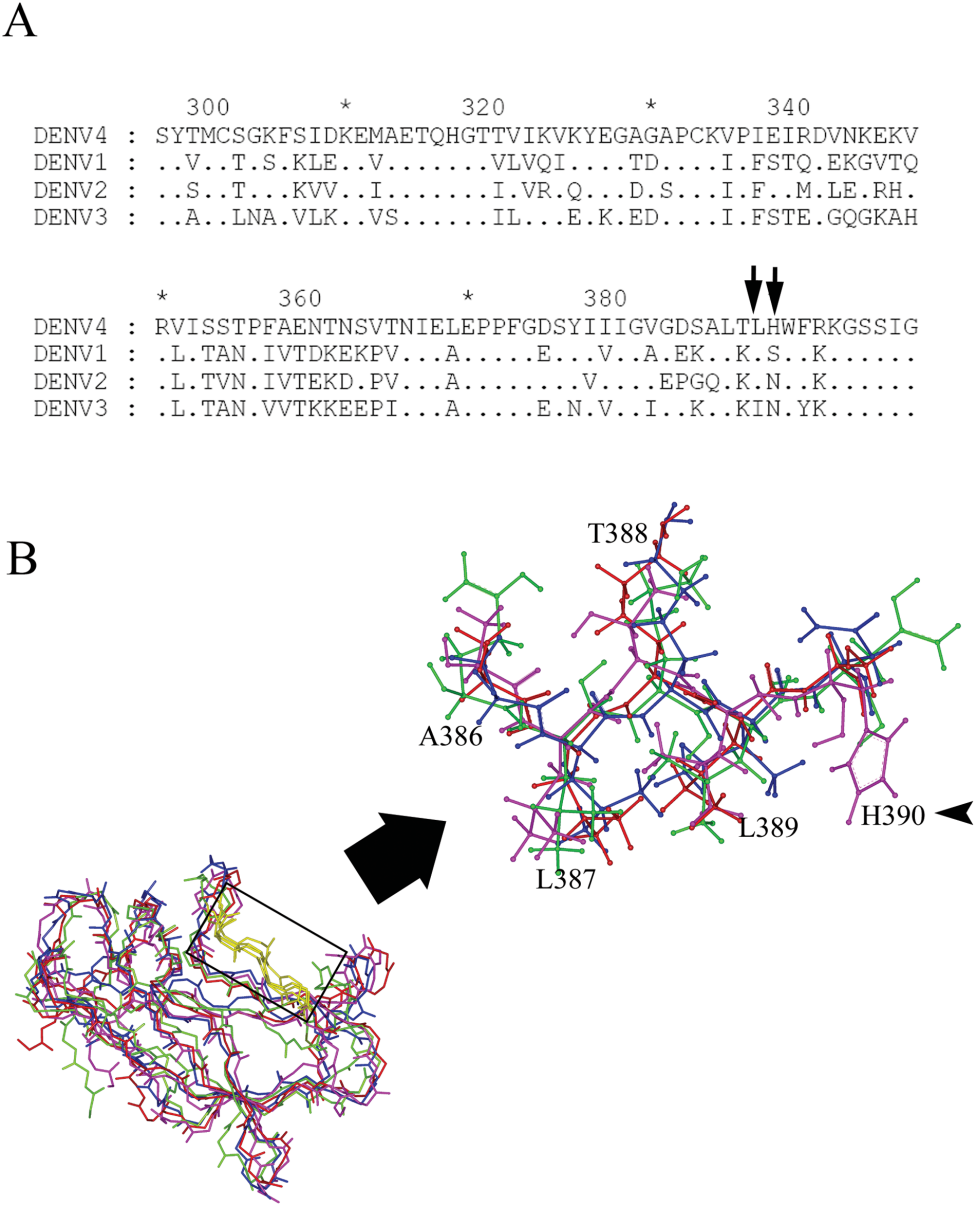
Amino acid sequence alignment of domain III from DENV1-4 and location of the mAb 1G6 epitope. **(A)** Comparison of amino acid sequences of ED3 proteins of wild-type DENV4 and other DENV serotypes. Identical amino acids were indicated by dots, and the unique residues were marker by arrows. **(B)** Overlay of crystal structures of DENV1-4 ED3. The localization of amino acid substitutions at positions 387–390 in the 3-D structure of monomeric DENV4 ED3, is shown from the side.

To understand the disparity in binding potential of the DENV4-specific mAb 1G6 among different serotypes, the amino acid sequences and crystal structures of DENV1-4 ED3 were aligned and analyzed (Fig 6). In the result of sequences alignment, although residues L387 and L389 are highly conserved among four DENV serotypes, the charges of critical residues T388 and H390 are unique compared with the corresponding residues of other three serotypes (Fig 6A), which is consistent with the inability of mAb 1G6 to bind DENV1-3.

In 3-D structures, the mAb 1G6 epitope is exposed on the surface of DENV4 ED3 protein (Fig 6B). The overlay result of crystal structures of DENV1-4 ED3 showed that the backbones of ED3 are highly similar among four DENV serotypes; however the critical residue H390 with an imidazole ring exposed has the most different side chain from its neighboring residues (Fig 6B). The result suggested that besides variations in charge, the subtle structure differences might also play an important role.

### The 1G6 epitope is highly conserved within and between different genotypes

To further discover whether the 1G6 epitope is variable within and between different genotypes, a total of 30 unique DENV4 ED3 protein sequences, including all four genotypes (I, II, III, and sylvatic), were downloaded from GenBank [35–37]. Sequences alignment showed that the antigenic region at which the 1G6 epitope located was completely conserved among all strains within and between different DENV4 genotypes (S2 Fig).

**Fig 7:**
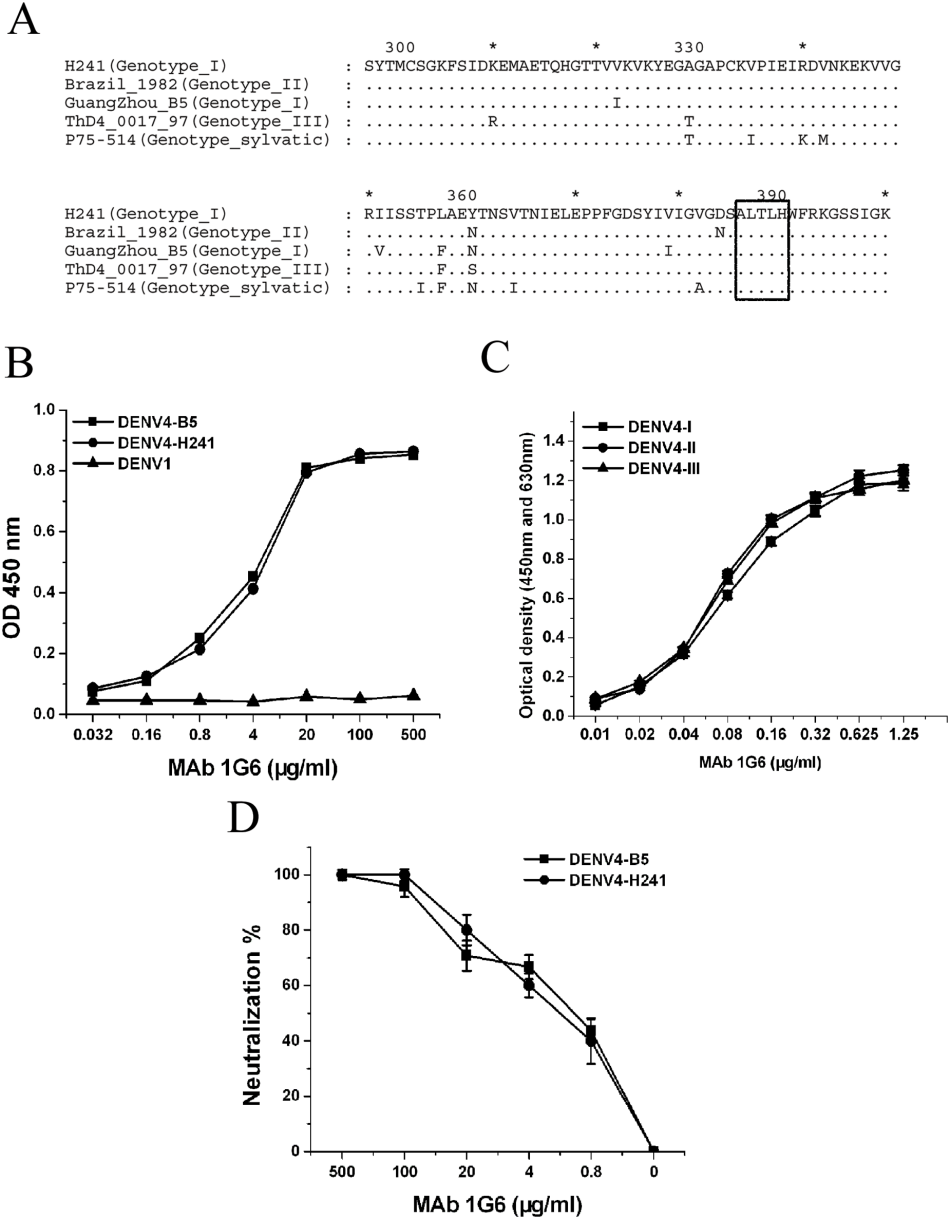
The epitope of mAb 1G6 is highly conserved within and between different genotypes. **(A)** Comparison of amino acid sequences of representative ED3 of four DENV4 genotypes. Identical amino acids were indicated by dots. **(B-C)** Binding of 1G6 with different DENV4 strains within genotype I **(B)** and representative recombinant ED3 between genotypes **(C)**. MAb binding was detected by ELISA. **(D)** Neutralization assay of 1G6 against DENV B5 and H241 strains.

The DENV4 strain H241 and strain B5 (genotype I) were further used to compare both the binding affinity and neutralizing ability within the same genotype (Fig 7A). The results showed that naturally occurring amino acid variation on DENV4 ED3 within the same genotype did not influence the binding of mAb 1G6 (p>0.05) by ELISA (Fig 7B). Consistent with the binding affinity, there was no difference between the neutralization titers (p>0.05, Fig 7D).

To test whether amino acid variances between different genotypes would influence the binding of mAb 1G6, affinity measurement by ELISA using rED3s from three DENV4 genotypes (genotype I, II and III) was conducted. The result showed that 1G6 bound to representative ED3 proteins from three genotypes with similar affinity (p>0.05) (Fig 7C), implying that mAb 1G6 perhaps has the potential to neutralize all DENV4 genotypes.

## Discussion

Recent studies have generated a great many DENV type-specific mAbs and identified many type-specific neutralizing epitopes on ED3 for DENV1-4. Although the crystal structure of the DENV4 ED3 protein has been solved, there have been few studies on fine mapping of type-specific neutralizing epitopes on the DENV4 ED3 protein [29]. In the present study, a panel of DENV type-specific, complex and sub-complex reactive mAbs were generated by immunizing mice with tandem ED3 protein. Amongst these mAbs, a novel DENV4-specific neutralizing mAb 1G6 was further characterized. This antibody can efficiently neutralize DENV4 at a PRNT_50_ of 1.52 ± 0.63 μg/ml *in vitro*, primarily by hindering DENV4 infection at a step before adsorption. Meanwhile, this antibody can confer protection against lethal DENV4 challenge *in vivo* even at 24 h after infection. The PRNT_50_ is comparable to that of complex and sub-complex neutralizing mAbs 4E11, 9F12 and 9D12 with PRNT_50_ of >2.4 μg/ml, 2×10^−8^ M (about 3 μg/ml) and 2 μg/ml against DENV4, respectively.

It was reported that most ED3-specific antibodies functioned by the blocking attachment of the virus to the cell receptor [38]; however, some ED3-specific antibodies have also been found to function at a step after virus attachment [39,40]. In our study, pre- and post- adsorption inhibition assay suggested that mAb 1G6 hinders an early step in the virus life cycle, most likely viral adsorption. It probably recognizes an epitope that are engaged in virus binding with cell receptors, which is consistent with the previous finding that ED3 is characteristic of many cell receptors [14].

**Fig 8:**
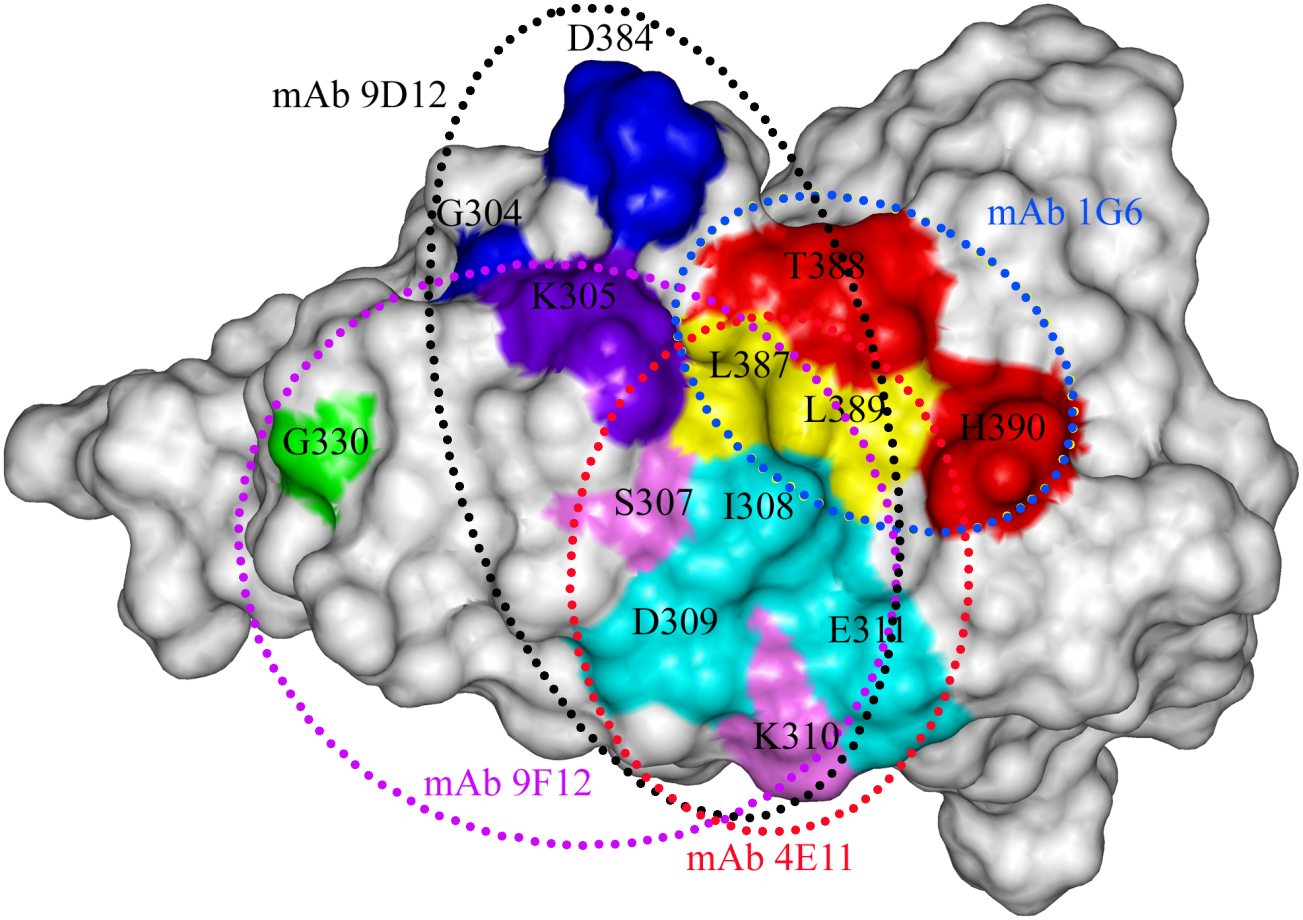
Relationship of 1G6 epitope with the already mapped complex and sub-complex neutralizing epitopes. The rings represent the area mapped to be the footprint of each mAb on the surface of ED3. The 4E11 epitope is shown in red and includes residues 307–312, 387, 389 and 391. The 9D12 epitope is shown in black and includes residues G304, K305, K307, K310 and P384. The epitope recognized by mAbs 9F12 is shown in purple and includes residues 305, 307, 310 and 330. The epitope for mAb 1G6 is shown in green and includes residues T388 and H390. The diagram is based on the crystal structure of the DENV4 ED3 protein (PDB ID 2H0P).

Previous studies have reported several complex and sub-complex neutralizing antibodies that recognize a set of overlapping epitopes that form a large antigenic area on ED3 centering on the A strand at residues 305–312. For instance, complex neutralizing mAb 4E11 (raised against DENV1 and can neutralize DENV1-4) recognized an epitope including residues 307–312, 387, 389 and 391 on DENV1 ED3, by alanine-scanning mutagenesis. Similarly, the epitope of complex mAb 9F12 (raised against DENV2) was mapped by yeast display to residues 305, 307, 310 and 330 on DENV2 ED3. Furthermore, a sub-complex neutralizing mAb 9D12 recognized an epitope composed of residues G304, K305, K307, K310 and P384 on DENV2 ED3 [26–28]. The epitope of DENV4 type-specific mAb 1G6 was compared with three sub-complex and complex-reactive epitopes on a 3-D structure of DENV4 ED3 [27,28,41]. As is shown in Fig 8, those DENV complex epitopes overlap with each other and center on the critical residues 305–312. The epitope region (^**387**^LTLH^**390**^) of 1G6 is spatially close to the large overlapping antigenic area and overlaps with the complex neutralizing epitope of mAb 4E11 at the residues of L387 and L389, but the two critical residues (T388 and H390) locate at the outside of the epitope region of mAb 4E11. Recently, a study by Sukupolvi-Petty et al. [29] generated a series of DENV4 type-specific neutralizing mAbs with their epitopes mapped to residues M301, K310, K325, A331, E338, V347, P356, T361, D375, Y377, H390 and F392 on the DENV4 ED3 protein, respectively; while other cross-reactive neutralizing mAbs recognized residues M301, E338, V347, V348, R350, P356 and L387. Our results showed that mAb 1G6 recognizes a unique epitope region compared to any of the already defined DENV4 neutralizing epitopes.

Dramatic surface differences in crystal structures of DENV1-4 ED3 among different serotypes have been showed by previous studies [42]. In the present study, our results showed clearly that although strand β9 is nearly consistent across ED3 structures of all four DENV serotypes, DENV4 indeed displays a unique surface in this region for antibodies to bind. Furthermore, the results of sequence alignment and crystal structure analysis showed that there are both charge and structure differences in the mAb 1G6 epitope region, indicating that both of these factors might responsible for its binding specificity. In the point mutation analysis of rED3, corresponding amino acid was substituted to glycine, which is known as a hydrophobic amino acid. In contrast to A (386), L (387) and L (389), which are also hydrophobic, T (388) and H (390) are polar amino acids. The change of polarity might also contribute to the reduction of the affinity.

In previous studies, amino acid 390 in the envelope glycoprotein of DENV2 was implicated to be a neurovirulent residue and controlled the replicative efficiency of DENV2 in cultured cells [43,44]. Complex neutralizing mAb 4E11, which interacts with the neighbouring residues of 390 in positions 389 and 391, might neutralize DENV by blocking the essential function of residue 390 through steric hindrance [41]. In our study, mAb 1G6 directly recognizes the residue 390 and its neighboring residues in DENV4 envelope protein, indicating that the residue 390 might also be very important for DENV4. However, the exact functions of the residue H390 on the neurovirulence and replication of DENV4 remain to be determined.

Dengue infection is thought to induce lifelong immunity against the same virus serotype [45]; while cross-reactive antibodies could increase the risk of developing more severe clinical outcomes of DENV infection via antibody-dependent enhancement (ADE) [46]. Furthermore, there is considerable genetic diversity within each serotype such that each has been subdivided into genotypes [47,48]. Previous reports have shown that naturally occurring strain variations within each serotype may limit the efficacy of antibody therapy or tetravalent vaccines against DENV as neutralization potency generally correlated with a narrowed genotype specificity [22,24]. For instance, naturally occurring mutations in ED3 between DENV3 genotypes could lead to a total loss of mAb binding. For DENV4, four major genotypes have been described, with sequence identity among all the DEN–4 virus strains ranging from 92.9 to 98.0%[35,36]. However, the epitope of mAb 1G6 located at a highly conserved region among different DENV4 strains of four genotypes. Also, mAb 1G6 bound representative ED3 proteins within and between DENV4 genotypes with the same affinity, indicating that naturally occurring variations outside the epitope region did not lead to altered antibody interactions. The results implied that mAb 1G6 could perhaps have the potential to neutralize all DENV4 genotypes. Inclusion of this epitope might be a good consideration for an effective vaccine.

In summary, we generated a novel DENV4 type-specific neutralizing antibody with both prophylactic and potent therapeutic efficacies in vivo and defined a novel type-specific neutralizing epitope region within DENV4 ED3. This epitope was shown to be highly conserve among and between different DENV4 genotypes. This antibody provides us with a good candidate for deep into the mechanism to neutralize DENV4 and the information obtained from this report on the type-specific monoclonal antibody will be useful for further development of dengue vaccines as well as antibody therapy.

## Supporting Information

S1 Fig Reactivity of the mAbs with respective DENV1, 2, 3 and 4 GST-tagged rED3 proteins by ELISA. The error bars represent the standard deviation.
(TIF)

S2 Fig Comparison of amino acid sequences of ED3 proteins of different DENV4 genotypes. In the sequence alignment, a dot indicates an identical amino acid compared with the strain Brazil 1982. The black box corresponds to the epitope region of mAb 1G6.
(TIF)

S1 Table Reactivity of monoclonal antibodies with four dengue virus serotypes by IFA. (DOC)
