# Supplementary figures and images for: Characterization of a Novel Dengue Serotype 4 Virus-Specific Neutralizing Epitope on the Envelope Protein Domain III
Source: PLoS One. 2015 Oct 2;10(10):e0139741. doi: 10.1371/journal.pone.0139741 (PMC4592203; doi:10.1371/journal.pone.0139741)

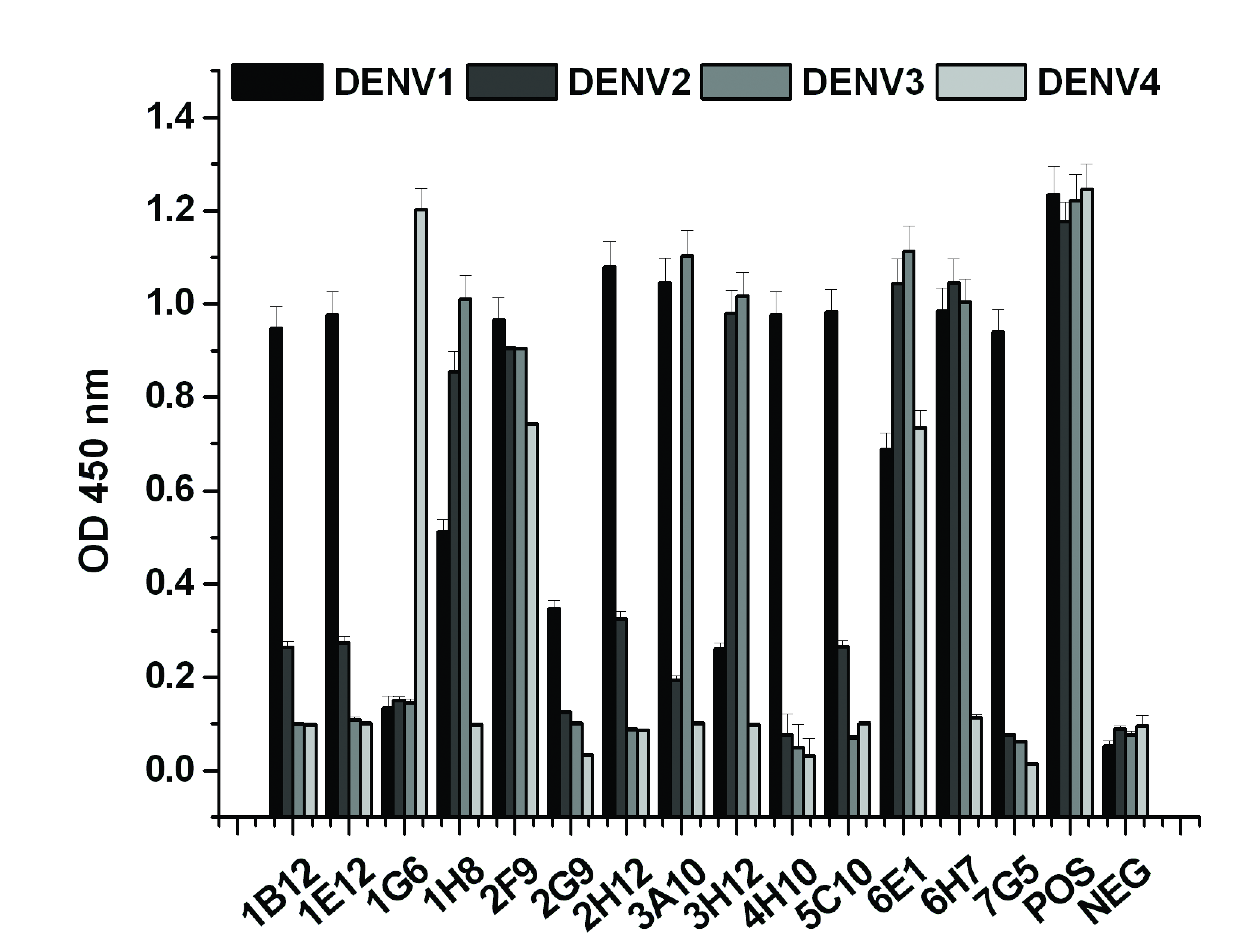

Supplement: S1 Fig — The error bars represent the standard deviation. (TIF) [file pone.0139741.s001.tif]

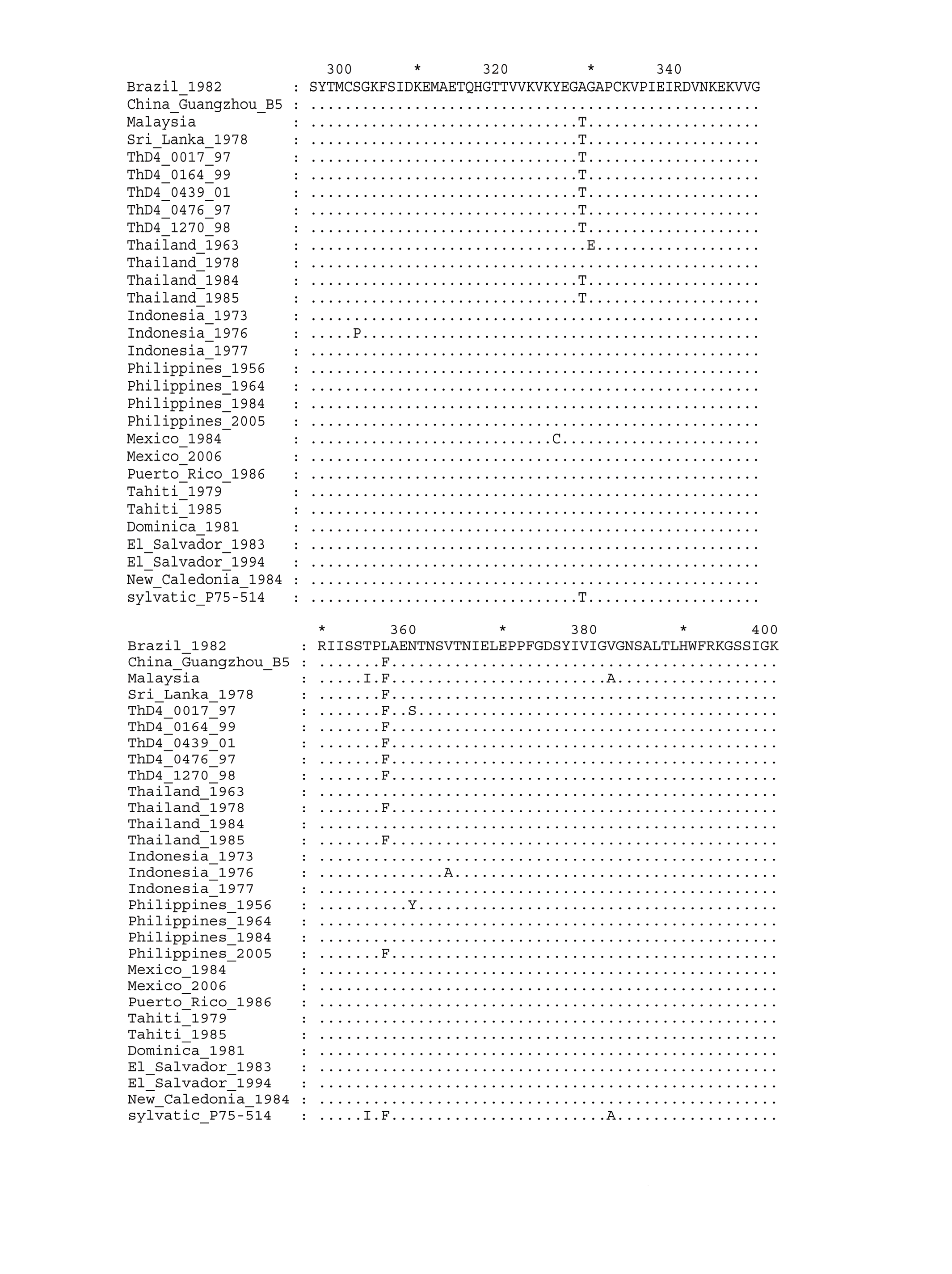

Supplement: S2 Fig — In the sequence alignment, a dot indicates an identical amino acid compared with the strain Brazil 1982. The black box corresponds to the epitope region of mAb 1G6. (TIF) [file pone.0139741.s002.tif]
