# Supplementary material for: Characterization of a Novel Dengue Serotype 4 Virus-Specific Neutralizing Epitope on the Envelope Protein Domain III
Source: PLoS One. 2015 Oct 2;10(10):e0139741. doi: 10.1371/journal.pone.0139741 (PMC4592203; doi:10.1371/journal.pone.0139741)
Supplement: S1 Table — (DOC) [file pone.0139741.s003.doc]

**Table S1 Reactivity of monoclonal antibodies with four dengue virus serotypes by IFA**.

| **mAb strains** | **Reactivity by IFA** | | | |
| --- | --- | --- | --- | --- |
|  | **DENV1** | **DENV2** | **DENV3** | **DENV4** |
| **1B12** | **+++** | **-** | **-** | **-** |
| **1E12** | **+++** | **-** | **-** | **-** |
| **1G6** | **-** | **-** | **-** | **+++** |
| **1H8** | **-** | **-** | **++** | **-** |
| **2F9** | **-** | **-** | **++** | **-** |
| **2G9** | **-** | **-** | **++** | **-** |
| **2H12** | **+++** | **-** | **-** | **-** |
| **3A10** | **+++** | **-** | **-** | **-** |
| **3H12** | **++** | **++** | **-** | **-** |
| **4H10** | **+++** | **++** | **+** | **-** |
| **5C10** | **++** | **-** | **-** | **-** |
| **6E1** | **++** | **++** | **++** | **+** |
| **6H7** | **++** | **-** | **-** | **-** |
| **7G5** | **+++** | **-** | **-** | **-** |
